# A Count Model to Study the Correlates of 60 Min of Daily Physical Activity in Portuguese Children

**DOI:** 10.3390/ijerph120302557

**Published:** 2015-02-26

**Authors:** Alessandra Borges, Thayse Natacha Gomes, Daniel Santos, Sara Pereira, Fernanda K. dos Santos, Raquel Chaves, Peter T. Katzmarzyk, José Maia

**Affiliations:** 1 CIFI^2^D, Faculty of Sports, University of Porto, Rua Dr. Plácido Costa, 91, 4250-Porto, Portugal; E-Mails: borges_alessandra@hotmail.com (A.B.); thayse_natacha@hotmail.com (T.N.G.); d.monteiro.santos13@gmail.com (D.S.); sara.s.p@hotmail.com (S.P.); fernandak.santos@hotmail.com (F.K.S.); 2 Department of Physical Education and Sports Science, CAV, Federal University of Pernambuco, 55608-680 Vitória de Santo Antão, PE, Brazil; 3 Physical Education Department, Federal University of Technology-Parana, Campus Curitiba, Curitiba/PR, 3165—Rebouças CEP 80230-901, Brazil; E-Mail: raquelnichele@live.com.pt; 4 Pennington Biomedical Research Center, Louisiana State University System, Baton Rouge, LA 70808, USA; E-Mail: Peter.Katzmarzyk@pbrc.edu

**Keywords:** physical activity, children, ISCOLE, MVPA

## Abstract

This study aimed to present data on Portuguese children (aged 9–11 years) complying with moderate-to-vigorous physical activity (MVPA) guidelines, and to identify the importance of correlates from multiple domains associated with meeting the guidelines. Physical activity (PA) was objectively assessed by accelerometry throughout seven days on 777 children. A count model using Poisson regression was used to identify the best set of correlates that predicts the variability in meeting the guidelines. Only 3.1% of children met the recommended daily 60 min of MVPA for all seven days of the week. Further, the Cochrane–Armitage chi-square test indicated a linear and negative trend (*p* < 0.001) from none to all seven days of children complying with the guidelines. The count model explained 22% of the variance in meeting MVPA guidelines daily. Being a girl, having a higher BMI, belonging to families with higher income, sleeping more and taking greater time walking from home to a sporting venue significantly reduced the probability of meeting daily recommended MVPA across the seven days. Furthermore, compared to girls, increasing sleep time in boys increased their chances of compliance with the MVPA recommendations. These results reinforce the relevance of considering different covariates’ roles on PA compliance when designing efficient intervention strategies to promote healthy and active lifestyles in children.

## 1. Introduction

Despite some controversy about a putative decline in children’s physical activity (PA) during the last few decades [1,2], it is generally accepted that children around the globe are currently failing to meet the World Health Organization (WHO) PA guidelines [3]. According to Strong *et al.* [4] and WHO recommendations, children and youth aged 5–17 years should accumulate at least 60 minutes of moderate-to-vigorous PA (MVPA) on a daily basis in order to enjoy the associated health benefits [3]. These recommendations are believed to prevent the development of chronic disease risk factors during childhood [5], which have been shown to track from childhood into adulthood [6].

Previous studies have reported mixed results with respect to the proportion of children complying with the recommended guidelines of at least 60 minutes of MVPA per day, which have ranged from 5% to 97% [7,8,9]. This large variation has been attributed to specific characteristics of the population studied, such as culture and demographics, as well as to different analysis procedures.

A comprehensive investigation of variation among children’s daily PA has identified a broad spectrum of correlates [10,11]. Three review papers [10,11,12] identified sub-sets of variables that play important roles in explaining differences in children’s PA levels; in addition, mixed results have been reported in terms of correlations and regression coefficients (magnitude and direction). Furthermore, distinct conceptual models and diverse statistical approaches have been used to investigate the existing relationships between PA levels and patterns and their recognized covariates [13,14]. For instance, parental social support has been shown to be positively associated with children’s PA, namely for two distinct sets of reasons: (i) purchasing equipment/payment of fees and transportation; and (ii) doing the activity with and watching/supervision [15]. Furthermore, recent evidence indicates that sleep time is associated with MVPA, suggesting that it plays a role on children’s behaviors towards achieving an active lifestyle [16]. Further, the influence of the built environment is now recognized to be one factor keeping kids away from an active lifestyle [17].

A commonly-used research template is the ecological model of four domains of active living [18] in which PA behavior is based, to some extent, on intra-personal, familial, social, cultural, environmental and policy characteristics. For instance, socioeconomic factors [19], distance to sports facilities [20] and time spent outdoors [10] have been shown to be predictors of PA habits during childhood. As such, in this study, we applied a Poisson regression model, aiming to identify the importance of biological, demographic, social, psychological, environmental and behavioral correlates in Portuguese children attaining MVPA recommendations. Prior to this model fitting process, data on the frequency of children attaining the MVPA recommendations will be presented. We hypothesize: (i) that the prevalence of children attaining the MVPA guidelines on all days is low; (ii) that girls will be less prone to comply with the guidelines; and (iii) that a set of environmental variables will significantly influence MVPA in children.

## 2. Materials and Methods

### 2.1. Sample

This study is part of the International Study of Childhood Obesity, Lifestyle and the Environment (ISCOLE), a multi-national investigation aiming to determine the relationships between lifestyle behaviors and obesity using the ecological approach as a research template that has been previously described [21]. The Portuguese ISCOLE sample comprises 777 5th grade Portuguese children, aged 9 to 11 years (358 boys), from 23 schools of the northern region of Portugal, in which 30 to 40 children were randomly selected; 50% of each sex was chosen, and the response rate was 95.7%. Written consent was obtained from each participant’s legal guardian and school directors. Further, this project was generally approved by the Pennington Biomedical Research Center Institutional Review Board and locally by the Ethics Committee of the University of Porto. Data quality control was systematically assessed and certified by the ISCOLE Coordinating Center [21].

### 2.2. Study Location Socio-Demographic Characteristics

Portugal is located in Southwest Europe, bordered by the Atlantic Ocean and Spain. The population comprises 10,603,800 inhabitants, from which 61.6% live in urban areas [22]. Life expectancy is 79.7 years, and the GDP per capita is US$21,558, which classifies it as a high income country [23,24]. In addition, the Porto metropolitan area has a population of 1,287,256 inhabitants, with a population density of 1,580.3 per/km^2^ and a population life expectancy of 78 years [25,26].

### 2.3. Anthropometry

Height, weight, sitting height and body fat were measured according to standardized ISCOLE procedures [21]. Each child was measured twice, and if there was a discrepancy between the two measurements beyond the tolerated error, a third measurement was taken. For the present analysis, the mean value of the closest two values for each measurement was used. Body mass index (BMI) was computed using the standard formula (weight (kg)/height (m)^2^), and WHO cut points using BMI percentiles [27] were used to classify children as normal weight, overweight or obese.

### 2.4. Maturity Offset

Biological maturation was indirectly estimated with the maturity offset procedure proposed by Mirwald *et al.* [28], which uses regression equations specific for both boys and girls using individual age, height, weight, sitting height and leg length. This procedure estimates the timing of the occurrence of peak height velocity (PHV), computes the distance each subject is from PHV and expresses it in decimal years. A positive (+) maturity offset represents the number of years a child is beyond PHV, whereas a negative (–) maturity offset represents the number of years he or she is before PHV.

### 2.5. Physical Activity

Children’s PA was objectively measured with the Actigraph GT3X+ accelerometer. The accelerometer was worn at the waist on an elasticized belt on the right mid-axillary line. ISCOLE children were encouraged to wear the accelerometer 24 h per day for at least 7 days (plus an initial familiarization day and the morning of the final day), including weekend days. Accelerometry has previously been shown to be a valid and reliable resource to track PA during childhood [29]. The minimal amount of accelerometer data that was considered acceptable for inclusion in the ISCOLE sample was 4 days with a minimum of 10 h of waking wear time per day, including at least one weekend day. Accelerometer non-wear time (any sequence of at least 20 consecutive minutes of zero activity counts) [30] was estimated after the sleep episode time was calculated using a published automated algorithm [31,32]. After this exclusion criteria (valid accelerometer information), the sample size reduced to 686 children with at least 4 valid accelerometer days (average daily wear time = 15.15 h); 582 had valid information for 7 days. Given that using only children with 7 valid days would imply a loss of about 25% of the initial sample, it was decided not to exclude subjects with 1 to 3 missing days (those with 4, 5 and 6 valid days). The missing data were estimated using a single imputation approach with the Expectation-Maximization (EM) algorithm, as previously advocated [33]. This was done in SPSS 21.

For the purpose of this study, our dependent variable comprises the number of days during a full week that each child attains 60 min of MVPA. MVPA was defined as all activities greater than 574 counts per 15 s [34]. This cut-point was chosen because it has been shown to be the most accurate to classify PA intensity in children when compared to indirect calorimetry [35].

### 2.6. Neighborhood and Home Environment Questionnaire

A questionnaire about the neighborhood and home environments was completed by all parents [21]. The questionnaire provides information on basic demographics, ethnicity, family health, socioeconomic factors, the home social environment, the home and neighborhood PA environment and the neighborhood built environment. For the present study, the neighborhood and home environment variables used included: family annual income, social factors (such as perceived crime rate in the neighborhood) and neighborhood context (such as sports facilities, public spaces for PA). A detailed description can be found in Katzmarzyk *et al*. [21].

### 2.7. Lifestyle Questionnaire

A questionnaire including behavioral and psychological information about sleep time, screen time, time spent on activities outside the home and children’s perceived parental support was completed by all children as detailed in Katzmarzyk *et al.* [21]. Screen time was computed based on the child-reported time spent watching television, playing video games or using the computer for leisure on weekdays and weekend days. The number of children fulfilling the recommendations of less than or equal to two hours of screen time/day [36] was also calculated.

### 2.8. Statistical Analysis

Exploratory data analysis and descriptive statistics were performed in SPSS 21. Since our dependent variable comprises the number of days during a full week that each child attains 60 min of MVPA, which in itself is a count, a Poisson regression model was used [37,38,39], as implemented in NCSS9 9 [40] software following the classical text of Cameron and Trivedi [41]. Further, a search technique (hierarchical forward selection with switching) implemented in the NCSS9 software 9 (NCSS9 Manual pp. 325–340) was used to find the smallest subset of the available regressor variables that predicts the number of days in which the child meets MVPA guidelines. These analyses were conducted in NCSS 9 [40]. The Cochrane–Armitage chi-square test, implemented in PEPI 4.0 [42] software, was used to identify a trend in MVPA 60 min counts (from never = 0 to everyday = 7).

## 3. Results

Table 1 presents the sample means and standard deviations for relevant variables by sex. On average, children have not experienced their PHV, as the maturity offset is −1.9 ± 0.89. Further, no significant differences were found between boys’ and girls’ weight (*p* = 0.74), height (*p* = 0.95) and BMI (*p* = 0.61), but girls were closer to their predicted PHV than boys (*p* < 0.001).

**Figure 1:**
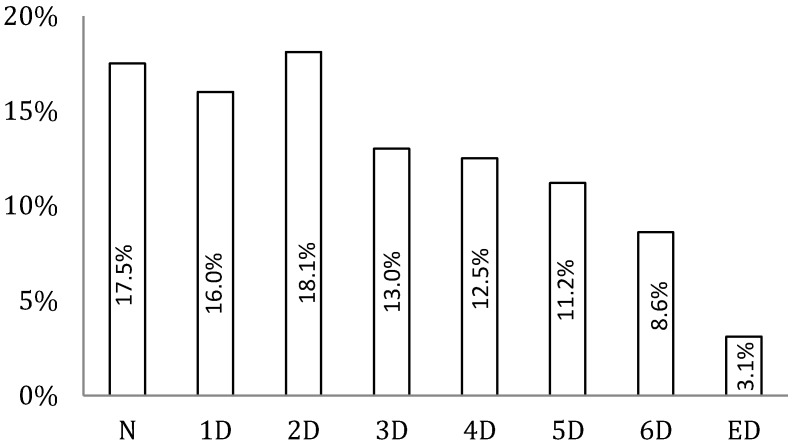
Percentage of children meeting the 60 min of MVPA guidelines by the number of days per week (N = never, 1D = 1 day, 2D = 2 days, 3D = 3 days, 4D = 4 days, 5D = 5 days, 6D = 6 days, ED = every day).

Only 3.1% of children met the recommendation of 60 min of MVPA for all seven days of the week, and 17.5% failed to meet this recommendation on any of the seven days (Figure 1). Further, the Cochrane–Armitage chi-square test indicated a linear and negative trend (χ^2^ = 94.60, *p* < 0.001) from none to seven days of children complying with the 60 min guideline.

**Table 1:** Boys’ and girls’ descriptive statistics (means (M) ± standard deviations (SD)), *t*- and *p*-values for testing sex differences.

Variable	Boys (M ± SD)	Girls (M ± SD)	*t*	*p*-Value	Total (M ± SD)
Height (cm)	143.5 ± 6.4	143.5 ± 7.1	0.060	0.952	143.5 ± 6.8
Weight (kg)	40.5 ± 9.2	40.3 ± 9.2	−0.332	0.740	40.4 ± 9.2
BMI (kg·m^−2^)	19.5 ± 3.5	19.4 ± 3.4	−0.511	0.610	19.5 ± 3.4
Maturity offset	−2.7 ± 0.4	−1.2 ± 0.5	41.028	<0.001	−1.9 ± 0.89

Table 2 presents data on biological, demographic, social, psychological, environmental and behavioral variables associated with PA during childhood. Most families had an annual income between €6,000–€11,999 (28.0%) and €12,000–€17,999 (26.5%); 41.8% of children reported being encouraged by parents to practice PA every day, whereas only 4.7% reported no support at all. However, only 11.1% of the children were driven to PA facilities by a parent every day. Further, 29.4% of our sample reported completely agreeing with the possibility of asking parents to practice PA with them.

**Table 2:** Summary information regarding individual items from biological, demographic, social, psychological, environmental and behavioral factors.

Factors	Frequency (%) or Mean
**Annual income**	
≤€6000	15.0
€6000–€11,999	28.0
€12,000–€17,999	26.5
€18,000–€23,999	13.3
€24,000–€29,999	6.1
€30,000–€35,999	4.2
€36,000–€41,999	2.6
≥€42,000	4.2
**Family support**	
**Encourage**	
Never	4.7
1–2 days	23.0
3–4 days	19.8
5–6 days	10.6
Every day	41.8
**Provide transport**	
Never	20.4
1–2 days	43.0
3–4 days	20.6
5–6 days	5.0
Every day	11.1
**I can ask my parent to do physically active things with me**	
Disagree a lot	13.7
Disagree a little	12.8
Disagree/agree	22.6
Agree a little	21.4
Agree a lot	29.4
**Time to walk from home to**	
**Indoor recreation**	
1–5 min	12.0
6–10 min	21.3
11–20 min	25.2
21–30 min	16.3
>30 min	11.7
Don’t know	13.6
**Bike/walking trail**	
1–5 min	12.5
6–10 min	12.1
11–20 min	14.4
21–30 min	15.6
>30 min	18.7
Don’t know	26.7
**Playing fields/courts**	
1–5 min	10.2
6–10 min	14.1
11–20 min	22.6
21–30 min	18.5
>30 min	13.8
Don’t know	20.7
**Public parks**	
1–5 min	10.5
6–10 min	18.1
11–20 min	25.9
21–30 min	16.5
>30 min	15.9
Don’t know	13.1
**There is a high crime rate in the neighborhood**	
Strongly disagree	43.4
Somewhat disagree	39.2
Somewhat agree	12.8
Strongly agree	4.5
**Time spent outside**	
Week days	
<1 h	39.7
1 h	32.8
2 h	20.3
3 h	5.7
4 h	0.6
5 or more	1.0
Weekend	
<1 h	30.3
1 h	23.3
2 h	21.7
3 h	12.7
4 h	5.0
5 or more	7.0
**Screen time**	
Week days	
≤2 h	73.6
>2 h	26.4
Weekend	
≤2 h	36.2
>2 h	63.8
**Sleep time (minutes (M ± SD))**	
Week days	517.2 ± 54.7
Weekend Days	562.8 ± 78.4

Most children spent a maximum of 20 min walking from their home to sports facilities (excluding “don’t know” answer), and most parents (82.6%) did not agree with the assertion that there is a high crime rate in their neighborhood. Nevertheless, the vast majority of the children (92.8%) did not spend more than two hours in activities outside the home during weekdays after school. During the weekend, this value decreased to 75.3%.

Almost 74% of the children met the recommendations of no more than two hours of screen time on weekdays, whereas during the weekend, only 36.2% met the recommendations. As for sleep time, children reported to have slept, on average, 517 and 563 minutes per day, during weekdays and weekend days, respectively.

Table 3 shows the Poisson regression results for the best subset selection using the forward covariate selection. The model pseudo-R^2^ (explained variance) was 22%. From the 12 putative covariates (see Table 2), only five plus the interaction of sex with sleep were statistically significant and entered the final model. Given that the beta coefficients are maximum likelihood estimates and difficult to interpret, their exponentiated values are expressed as rate ratios (RR), which makes them easier to understand. Girls, on average, comply with the MVPA guidelines only two days a week; this is the RR (exponentiation of the intercept (log of the expected count) when all predictors in the model are evaluated at zero). Compared to girls, boys were two-times (108%) more likely to comply with the 60 min MVPA guidelines. Overweight and obese children were less likely (about 12%) to comply with the recommendations compared to normal weight children. The same occurs to those belonging to families with higher income (a reduction of ~8%) compared to lower income families. For each category increase in the reported time it takes children to walk from home to an indoor recreation facility location, an expected decrease (~4.3%) in the number of MVPA counts across the seven days was found. Children who sleep more hours during the week reduced the expected number of complying days by 5%. Further, a sex-by-mean sleep time during the week was significant (RR = 1.125, 95% CI = 1.105 to 1.248). This suggests that, compared to girls, if boys increase, on average, 1, 2 or 3 hours of sleep during the week, they were more likely to increase their chances of complying with the MVPA recommendations by a factor of 2.35, 2.64 or 2.97, respectively.

**Table 3:** MVPA Poisson regression coefficients, standard errors (SE), *p*-values, rate ratios (RR) and corresponding 95% confidence intervals (95% CI).

Variables	Beta (SE)	*p*-Value	RR	95% RR CI
Intercept	0.907 (0.063)	<0.001	2.278	2.189 to 2.806
Sex	0.734 (0.048)	<0.001	2.083	1.895 to 2.291
BMI	−0.122 (0.046)	<0.001	0.927	0.901 to 0.954
AI	−0.075 (0.014)	<0.001	0.959	0.925 to 0.996
TIR	−0.043 (0.015)	0.004	0.957	0.928 to 0.986
SW	−0.152 (0.043)	<0.001	0.858	0.788 to 0.934
SSW	0.118 (0.052)	0.024	1.125	1.015 to 1.248

Notes: BMI, body mass index; AI, annual income; TIR, time to walk from home to indoor recreation facilities; SW, mean sleep time during the week; SSW, interaction of sex with mean sleep time during the week.

## 4. Discussion

Several studies have reported the prevalence of meeting PA guidelines in children and adolescents [1,13,14]. For example, Telford *et al.* [43] investigated MVPA patterns over a week and reported that, overall, 31% of boys and 16% of girls met MVPA recommendations in a longitudinal study of Australian children aged 8–12 years. Further, in a study of four consecutive days with accelerometers, Baptista *et al.* [44] found that, on average, 36% of Portuguese youth aged 10–11 years (boys = 51.6%, girls = 22.5%) were considered sufficiently active using the 60 min·d^−1^ of MVPA guidelines. On the contrary, data from seven European countries, including Belgium, Greece, Hungary, the Netherlands, Norway, Slovenia and Spain, showed that only 4.6% of the girls and 16.8% of the boys met the recommendations [45]. These results are in contrast with those from our sample in which only 3.1% of all children meet MVPA recommendations on all seven days of the week, and 17.5% did not attain the recommendations on any of the seven days (see Figure 1). Several factors, including population and sampling differences, MVPA definition and data processing protocols, are likely contributors to the differences found across studies. Furthermore, the existence of different intensity-related cut points for children’s and adolescents’ PA has hindered research efforts to quantify, understand and intervene on PA behavior [35]. For example, only the Telford *et al.* [43] study used the same MVPA cut off point during the seven days as the present study. Additionally, the differences found between Australian and Portuguese children may be attributable to cultural and behavioral differences.

When investigating children’s PA patterns, there is a tendency to compare weekdays and weekend days [43,46,47]. For instance, Telford *et al.* [43] concluded that in Australian children, Friday (boys = 39%, girls = 21%) and Sunday (boys = 16%, girls = 10%) were the highest and lowest compliant days of the week, respectively, for meeting recommendations. Ramirez *et al.* [47], using the same cut-off point as our study, showed that 26.6% and 24.5% of Liverpool (England) youths and 26.0% and 27.1% of Madrid (Spain) youths achieved the recommended levels of MVPA on weekdays and weekend days, respectively. In our case, the most compliant day was Friday (boys = 61.3%, girls = 37.0%), and the least was Sunday (boys = 28.5%, girls = 15%). Further, we identified a linear negative trend in compliance from one to seven days, which can be compared to the Crespo *et al.* [7] study in which, on average, children achieved 59 ± 23 min of MVPA per day across all seven days. However, when Crespo *et al.*’s [7] daily MVPA data were individually tabulated, only 24 children (21%) engaged in ≥60 min of MVPA on at least five days, and only six children (5%) achieved ≥60 min of MVPA on all seven days.

It has been postulated that distinct biological and demographic characteristics play important roles in governing children’s daily PA levels and patterns [10]. As such, it is important to consider a comprehensive set of these correlates, which are believed to explain the variation in children’s MVPA [11]. Our model explained 22% of the total variance in meeting MVPA guidelines, but only four correlates (sex, BMI, time to walk from home to indoor recreation facilities, mean sleep time during the week) and one interaction (interaction of sex with mean sleep time during the week) were statistically significant. As expected, sex was a significant predictor of MVPA, and being male doubled the chances of attaining the guidelines. This result is in line with previous reports [46] that concluded that sex was the most significant correlate of MVPA. A possible explanation for this result is that boys and girls engage in different types of activities with marked intensity differences [48]. For example, Blatchford *et al.* [49] showed that during school hours, boys are more likely to engage in ball games and vigorous activities, whereas girls are more prone to engage in quieter games without much physical contact. Another possible explanation might be attributable to maturational status. Thompson *et al.* [50] showed that girls mature earlier than boys, and PA tends to decrease with maturity, which might explain sex differences. However, maturity was not a significant MVPA predictor in the present study, which may be related to the narrow age range of the sample (nine to 11 years).

Being overweight/obesity was found to be negatively associated with the number of days children complied with MVPA recommendations, which is consistent with previous studies [50]. For example, Bergh *et al.* [51], studying 1,129 Norwegian 11-year-old children, found that those with normal weight scored higher on percentage daily MVPA than overweight/obese children. Yet, it is not always easy to clarify if having a high BMI leads children to be less physically active or if it is the other way around, *i.e.*, do less active children have a tendency to become heavier [52]? It has been observed that obese children, whom are less active, also present poorer motor skill proficiency as compared to their normal weight peers [53], lower physical competence perception and success, as well as lower peer acceptability in games and sports [48].

In our sample, being part of a higher income family reduces the likelihood of attaining the guidelines by ~8%. Nogueira *et al.* [19] studied the associations between children’s obesity, sports activity and perceived environmental characteristics with socioeconomic status and found that low and medium socioeconomic status children were more likely to be obese and less likely to participate in sports activity than their high socioeconomic status peers. This result seems to be in disagreement with ours. However, it has been suggested [54] that lower income neighborhoods provide a greater amount of government-funded opportunities for PA when compared to higher income neighborhoods. Furthermore, it needs to be acknowledged that sports activity is only one facet of overall PA, which may contribute to further explaining this discrepancy.

For each category increase in walking time from home to an indoor recreation facility, a 4.3% decrease in complying with MVPA guidelines is expected, which is in line with previous research [55,56]. In a recent review, Oliveira *et al.* [55] found a significant positive association between proximity to parks and playgrounds and children’s PA. Further, Tappe *et al.* [56] examined the association between parent reports of their neighborhood environment and children’s activity within the neighborhood and in parks and concluded that parent-reported proximity to play areas correlated positively with accelerometer outcomes. One of the factors that may explain this observation is active transportation, *i.e.*, children walk to places that are closer to their homes to engage in diversified PA. With increasing distances either of two behaviors are likely to occur: (i) children prefer not to practice PA in these places [57]; or (ii) children are forced to use other means of transportation [58].

When analyzing the results for BMI, family annual income and walking time from home to a sport venue, we hypothesize that there is a relationship between these three variables. An argument could be raised that annual income has a major influence on active transportation to sports facilities, as the higher the annual income, the greater availability of cars for transportation, leading to diminished levels of PA, which, in turn, promotes BMI increases.

In our model, sleep time was also negatively associated (~4%) with guidelines’ compliance. Data about the influence of sleep time in PA and, more particularly, in MVPA are still unclear. For example, in a very recent published report of a multicenter European study, Soric *et al.* [59] found that for every hour increase in sleep time, a 16 min decrease in MVPA was expected as assessed by means of an armband sensor. Similarly, Williams *et al.* [60] studying 3-, 5- and 7-year-old children concluded that those who are more physically active during the day have shorter total sleep time and spend more time awake at night than their less active peers. Further, Pesonen *et al.* [61] using data from 9 to 16 year-old children reported that the relationship between higher levels of PA and poorer sleep is bidirectional: one standard deviation (SD) unit increase in PA during the day means that sleep duration decreases by 0.30 SD units and sleep efficiency decreases by 0.16 SD units; while there is a one SD increase in sleep duration and efficiency for the preceding night, and PA for the following day increases by 0.09 and 0.16 SD units, respectively. Differently, Ekstedt *et al.* [16] found an association between MVPA and the sum (in minutes) of all sleep epochs between sleep onset and sleep end to be non-significant. Possible explanations for the results found in the present study can be related to the fact that, as suggested by Olds *et al.* [62], the hours of the day are limited, and sleeping longer reduces the time available for engagement in other activities. Additionally, Williams *et al.* [60] suggest that PA promotes better sleep rather than more sleep, and in this case, more physically active children should have better sleep, rather than a longer one, as compared to their less active peers. Sleep disorders have been linked with undesirable outcomes on children’s health [63], namely increased BMI, which may well be mediated by PA. If this were to be consistently proven, then community interventions aiming at tackling the obesity epidemic and the associated morbidities would largely benefit from this evidence.

The significant interaction found by sex and mean sleep time during the week suggests that increasing by 1, 2 or 3 hours boys’ mean sleep time increases their chances to achieve the MVPA guidelines, compared to girls, by factors of 2.35, 2.64 or 2.97, respectively. This result reveals that the effect of sleep time, on the MVPA, is moderated by sex, which can be taken into account when this relationship (PA and sleep time) is studied. Pesonen *et al.* [61] also found a moderated gender effect in this association, with stronger effects among girls.

Notwithstanding the significance of the present findings, some limitations must be addressed. Firstly, restricting the sample to the Porto urban area limits the generalization of the results in terms of the Portuguese population. Yet, a comparison of the present sample characteristics with available information from the Portuguese population of the same age and sex was done. For example, in data not shown here, no differences were found in the prevalence of overweight/obesity [64], as well as in the percentage of children attaining sufficient levels of PA [44]. Secondly, we must bear in mind that we used Evenson *et al.*’s cut-off points [34], which have been proven widely acceptable in MVPA classification accuracy [35], although the cut-off issue continues to be an unsolved matter. Thirdly, we used a single imputation approach with the EM algorithm to estimate the missing data. Although multiple imputation could be used, Cattellier *et al*. [33] have shown that both approaches performed equally well in terms of bias or precision. Fourthly, although the ISCOLE decision to consider a minimum of four days of valid accelerometry data may be debatable, we conducted a generalizability study with the Portuguese data, with a single facet (days), using the urGENOVA software [65], and found a G-coefficient of 0.82 for seven days, and 0.73 for four days. This shows that starting from four days (with a weekend day included), we have very reliable accelerometry information. Lastly, limiting our sample age to 9–11 years may be a restrictive factor, although it marks the beginning of a very important transition phase from late childhood to adolescence [66] and a significant change in school habits. On the other hand, the present study has several strong points. Firstly, the high quality dataset is a part of an international study with unparalleled information, as shown by Katzmarzyk *et al.* [21]. Secondly, the usage of objective PA measurement during seven consecutive days with a substantial time frame allows for a high degree of consistency to detect levels of daily PA over a week. Thirdly, the broad spectrum of environmental variables is unique in a single study.

## 5. Conclusions

In conclusion, the present results show that the percentage of children meeting the 60 min of daily MVPA recommendations, seven days a week, is low. Further, there is a linear negative trend in the compliance with the MVPA guidelines from one to seven days. In addition, the number of days children complied with MVPA guidelines is positively associated with sex (male), but negatively with BMI, family income, walking time from home to a sporting venue and mean sleep time during the week. More efforts are needed to uncover the relevancy of different environmental correlates on meeting PA recommendations. These results emphasize the importance of taking into account multiple levels of influence when developing interventions to promote healthy and active lifestyles.
